# (*E*)-Benzyl 3-(3-nitro­benzyl­idene)dithio­carbazate

**DOI:** 10.1107/S1600536809045723

**Published:** 2009-11-14

**Authors:** Huan-Qiu Li, Yin Luo, Xuan Qin, Hai-Liang Zhu

**Affiliations:** a State Key Laboratory of Pharmaceutical Biotechnology, Nanjing University, Nanjing 210093, People’s Republic of China

## Abstract

In the title compound, C_15_H_13_N_3_O_2_S_2_, the dihedral angle between the aromatic rings is 87.8 (2)°. In the crystal, inversion dimers occur linked by pairs of N—H⋯S hydrogen bonds.

## Related literature

For background to carbodithio­ates, see: Tarafder *et al.* (2002 ▶).
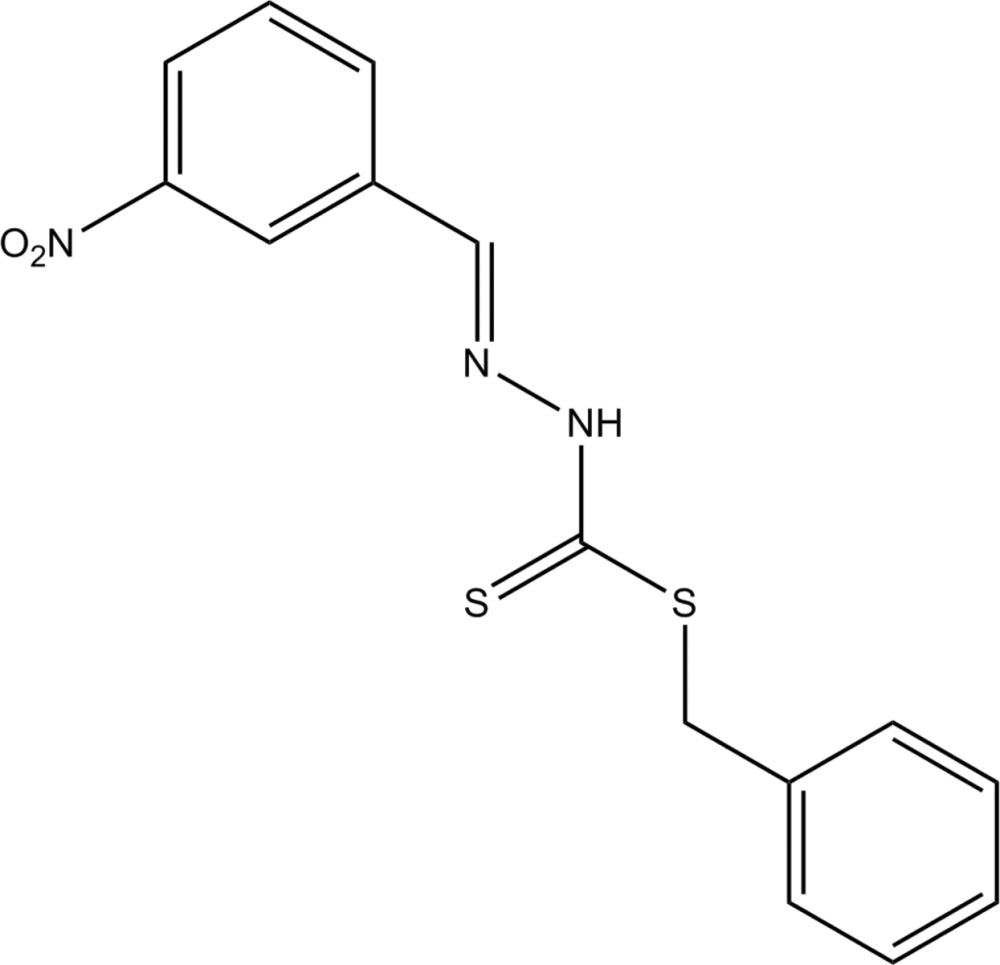


## Experimental

### 

#### Crystal data

C_15_H_13_N_3_O_2_S_2_
*M* *_r_* = 331.40
Monoclinic, 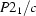

*a* = 5.2175 (10) Å
*b* = 26.213 (5) Å
*c* = 11.887 (2) Å
β = 90.67 (3)°
*V* = 1625.6 (6) Å^3^
*Z* = 4
Mo *K*α radiation
μ = 0.34 mm^−1^
*T* = 293 K
0.30 × 0.30 × 0.10 mm

#### Data collection

Enraf–Nonius CAD-4 diffractometer
Absorption correction: ψ scan (North *et al.*, 1968 ▶) *T*_min_ = 0.906, *T*_max_ = 0.967
9486 measured reflections
2788 independent reflections
951 reflections with *I* > 2σ(*I*)
*R*_int_ = 0.097
200 standard reflections every 3 reflections intensity decay: 1%

#### Refinement

*R*[*F* ^2^ > 2σ(*F* ^2^)] = 0.045
*wR*(*F* ^2^) = 0.106
*S* = 0.73
2788 reflections
211 parameters
H atoms treated by a mixture of independent and constrained refinement
Δρ_max_ = 0.15 e Å^−3^
Δρ_min_ = −0.16 e Å^−3^

### 

Data collection: *CAD-4 Software* (Enraf–Nonius, 1989 ▶); cell refinement: *CAD-4 Software*; data reduction: *XCAD4* (Harms & Wocadlo, 1995 ▶); program(s) used to solve structure: *SHELXS97* (Sheldrick, 2008 ▶); program(s) used to refine structure: *SHELXL97* (Sheldrick, 2008 ▶); molecular graphics: *SHELXTL* (Sheldrick, 2008 ▶); software used to prepare material for publication: *SHELXTL*.

## Supplementary Material

Crystal structure: contains datablocks global, I. DOI: 10.1107/S1600536809045723/hb5203sup1.cif

Structure factors: contains datablocks I. DOI: 10.1107/S1600536809045723/hb5203Isup2.hkl

Additional supplementary materials:  crystallographic information; 3D view; checkCIF report

## Figures and Tables

**Table 1:** Hydrogen-bond geometry (Å, °)

*D*—H⋯*A*	*D*—H	H⋯*A*	*D*⋯*A*	*D*—H⋯*A*
N1—H1*A*⋯S2^i^	0.93 (3)	2.45 (4)	3.365 (4)	170 (3)

Symmetry code: (i) 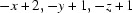.

**Fig. 1.**
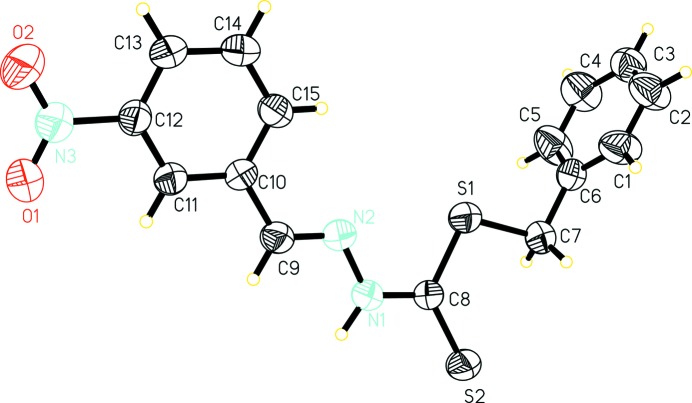
The structure of the title compound (I) showing 50% displacement ellipsoids.

## supplementary crystallographic information

### Experimental

The title compound was prepared by stirring a mixture of benzyl hydrazinecarbodithioate (396 mg, 2 mmol), 3-nitrobenzaldehyde (302 mg, 2 mmol) in methanol (10 ml) for 1 h. After keeping the filtrate in air for 7 d, yellow blocks of (I) were formed.

### Refinement

The N-bound N atom was located in a difference map and freely refined. The other H atoms were positioned geometrically (C—H = 0.93 Å for the aromatic H atoms and C—H = 0.96 Å for the aliphatic H atoms) and were refined as riding, with *U*_iso_(H) = 1.2*U*_eq_(C) and *U*_iso_(H) = 1.2*U*_eq_(N).

### Crystal data

**Table:**

C_15_H_13_N_3_O_2_S_2_	*F*(000) = 688
*M**_r_* = 331.40	*D*_x_ = 1.354 Mg m^−^^3^
Monoclinic, *P*2_1_/*c*	Mo *K*α radiation, λ = 0.71073 Å
Hall symbol: -P 2ybc	Cell parameters from 25 reflections
*a* = 5.2175 (10) Å	θ = 9–12°
*b* = 26.213 (5) Å	µ = 0.34 mm^−^^1^
*c* = 11.887 (2) Å	*T* = 293 K
β = 90.67 (3)°	Block, yellow
*V* = 1625.6 (6) Å^3^	0.30 × 0.30 × 0.10 mm
*Z* = 4	

### Data collection

**Table:**

Enraf–Nonius CAD-4 diffractometer	951 reflections with *I* > 2σ(*I*)
Radiation source: fine-focus sealed tube	*R*_int_ = 0.097
graphite	θ_max_ = 25.0°, θ_min_ = 1.9°
ω/2θ scans	*h* = −6→5
Absorption correction: ψ scan (North *et al.*, 1968)	*k* = −31→29
*T*_min_ = 0.906, *T*_max_ = 0.967	*l* = −14→14
9486 measured reflections	200 standard reflections every 3 reflections
2788 independent reflections	intensity decay: 1%

### Refinement

**Table:**

Refinement on *F*^2^	Primary atom site location: structure-invariant direct methods
Least-squares matrix: full	Secondary atom site location: difference Fourier map
*R*[*F*^2^ > 2σ(*F*^2^)] = 0.045	Hydrogen site location: inferred from neighbouring sites
*wR*(*F*^2^) = 0.106	H atoms treated by a mixture of independent and constrained refinement
*S* = 0.73	*w* = 1/[σ^2^(*F*_o_^2^) + (0.0448*P*)^2^] where *P* = (*F*_o_^2^ + 2*F*_c_^2^)/3
2788 reflections	(Δ/σ)_max_ < 0.001
211 parameters	Δρ_max_ = 0.15 e Å^−^^3^
0 restraints	Δρ_min_ = −0.16 e Å^−^^3^

### Special details

**Table:**

Geometry. All e.s.d.'s (except the e.s.d. in the dihedral angle between two l.s. planes) are estimated using the full covariance matrix. The cell e.s.d.'s are taken into account individually in the estimation of e.s.d.'s in distances, angles and torsion angles; correlations between e.s.d.'s in cell parameters are only used when they are defined by crystal symmetry. An approximate (isotropic) treatment of cell e.s.d.'s is used for estimating e.s.d.'s involving l.s. planes.
Refinement. Refinement of *F*^2^ against ALL reflections. The weighted *R*-factor *wR* and goodness of fit *S* are based on *F*^2^, conventional *R*-factors *R* are based on *F*, with *F* set to zero for negative *F*^2^. The threshold expression of *F*^2^ > σ(*F*^2^) is used only for calculating *R*-factors(gt) *etc*. and is not relevant to the choice of reflections for refinement. *R*-factors based on *F*^2^ are statistically about twice as large as those based on *F*, and *R*- factors based on ALL data will be even larger.

### Fractional atomic coordinates and isotropic or equivalent isotropic displacement parameters (Å^2^)

**Table:**

	*x*	*y*	*z*	*U*_iso_*/*U*_eq_	
C1	1.5564 (10)	0.38939 (18)	1.0058 (4)	0.1065 (16)	
H1	1.6613	0.3787	0.9477	0.128*	
C2	1.5833 (10)	0.36729 (18)	1.1102 (5)	0.1215 (18)	
H2	1.7070	0.3423	1.1222	0.146*	
C3	1.4312 (11)	0.38176 (19)	1.1951 (4)	0.0986 (14)	
H3	1.4491	0.3668	1.2658	0.118*	
C4	1.2549 (11)	0.4177 (2)	1.1770 (4)	0.1203 (18)	
H4	1.1474	0.4275	1.2349	0.144*	
C5	1.2307 (9)	0.44050 (17)	1.0726 (5)	0.1170 (17)	
H5	1.1095	0.4661	1.0616	0.140*	
C6	1.3817 (9)	0.42606 (17)	0.9862 (4)	0.0740 (11)	
C7	1.3604 (11)	0.4511 (2)	0.8717 (4)	0.0893 (15)	
C8	1.0763 (8)	0.45488 (13)	0.6740 (3)	0.0662 (10)	
C9	0.5747 (8)	0.38503 (16)	0.5578 (3)	0.0740 (12)	
H9	0.5412	0.4063	0.4967	0.089*	
C10	0.4214 (8)	0.33846 (15)	0.5727 (3)	0.0648 (10)	
C11	0.2307 (8)	0.32751 (16)	0.4958 (3)	0.0716 (11)	
H11	0.1940	0.3499	0.4371	0.086*	
C12	0.0946 (8)	0.28268 (18)	0.5071 (4)	0.0763 (12)	
C13	0.1352 (9)	0.24961 (16)	0.5946 (4)	0.0997 (15)	
H13	0.0389	0.2199	0.6014	0.120*	
C14	0.3244 (9)	0.26178 (18)	0.6727 (4)	0.1020 (15)	
H14	0.3563	0.2399	0.7329	0.122*	
C15	0.4658 (8)	0.30577 (16)	0.6623 (3)	0.0838 (12)	
H15	0.5917	0.3136	0.7156	0.101*	
H1A	0.851 (7)	0.4589 (13)	0.540 (3)	0.100 (15)*	
N1	0.8928 (7)	0.43995 (14)	0.6035 (3)	0.0779 (10)	
N2	0.7523 (7)	0.39663 (13)	0.6274 (3)	0.0739 (9)	
N3	−0.1075 (8)	0.27046 (17)	0.4218 (4)	0.0953 (12)	
O1	−0.1536 (6)	0.30218 (13)	0.3506 (3)	0.1166 (11)	
O2	−0.2120 (8)	0.22932 (15)	0.4285 (3)	0.1572 (16)	
S1	1.1060 (2)	0.41799 (4)	0.79464 (9)	0.0806 (4)	
S2	1.2592 (2)	0.50568 (4)	0.64490 (9)	0.0898 (4)	
H7A	1.508 (7)	0.4456 (14)	0.827 (3)	0.109 (17)*	
H7B	1.308 (8)	0.4842 (16)	0.871 (3)	0.14 (2)*	

### Atomic displacement parameters (Å^2^)

**Table:**

	*U*^11^	*U*^22^	*U*^33^	*U*^12^	*U*^13^	*U*^23^
C1	0.121 (4)	0.107 (4)	0.092 (4)	0.028 (3)	0.039 (3)	0.019 (3)
C2	0.128 (4)	0.128 (4)	0.109 (4)	0.051 (3)	0.027 (4)	0.034 (4)
C3	0.108 (4)	0.102 (4)	0.085 (4)	0.000 (3)	−0.004 (3)	0.005 (3)
C4	0.135 (5)	0.142 (5)	0.084 (4)	0.029 (4)	0.022 (3)	−0.002 (3)
C5	0.123 (4)	0.132 (4)	0.097 (4)	0.060 (3)	0.015 (3)	0.008 (3)
C6	0.070 (3)	0.076 (3)	0.076 (3)	−0.005 (2)	−0.005 (3)	−0.001 (3)
C7	0.077 (4)	0.109 (5)	0.082 (4)	−0.009 (3)	−0.010 (3)	0.016 (3)
C8	0.063 (3)	0.068 (3)	0.068 (3)	−0.002 (2)	0.001 (2)	0.007 (2)
C9	0.080 (3)	0.078 (3)	0.064 (3)	−0.005 (3)	0.002 (3)	0.005 (2)
C10	0.063 (3)	0.063 (3)	0.068 (3)	−0.006 (2)	0.006 (2)	0.000 (2)
C11	0.068 (3)	0.079 (3)	0.067 (3)	−0.007 (3)	0.003 (3)	0.005 (2)
C12	0.070 (3)	0.089 (4)	0.069 (3)	−0.012 (3)	−0.002 (3)	0.000 (3)
C13	0.103 (4)	0.089 (3)	0.107 (4)	−0.033 (3)	−0.017 (3)	0.027 (3)
C14	0.111 (4)	0.087 (4)	0.108 (4)	−0.020 (3)	−0.015 (3)	0.035 (3)
C15	0.094 (3)	0.078 (3)	0.079 (3)	−0.006 (3)	−0.013 (2)	0.012 (3)
N1	0.080 (3)	0.079 (3)	0.075 (3)	−0.010 (2)	−0.008 (2)	0.015 (2)
N2	0.076 (3)	0.068 (2)	0.077 (2)	−0.005 (2)	0.002 (2)	0.0053 (19)
N3	0.096 (3)	0.097 (4)	0.094 (3)	−0.010 (3)	−0.005 (3)	0.000 (3)
O1	0.114 (3)	0.127 (3)	0.108 (2)	−0.019 (2)	−0.031 (2)	0.016 (2)
O2	0.180 (4)	0.141 (3)	0.150 (3)	−0.082 (3)	−0.045 (3)	0.017 (3)
S1	0.0798 (8)	0.0879 (8)	0.0741 (7)	−0.0113 (6)	0.0010 (6)	0.0101 (6)
S2	0.0950 (9)	0.0826 (8)	0.0917 (8)	−0.0215 (7)	−0.0063 (6)	0.0142 (6)

### Geometric parameters (Å, °)

**Table:**

C1—C6	1.343 (5)	C9—N2	1.272 (4)
C1—C2	1.375 (5)	C9—C10	1.471 (5)
C1—H1	0.9300	C9—H9	0.9300
C2—C3	1.346 (5)	C10—C11	1.373 (4)
C2—H2	0.9300	C10—C15	1.384 (5)
C3—C4	1.332 (5)	C11—C12	1.380 (5)
C3—H3	0.9300	C11—H11	0.9300
C4—C5	1.381 (5)	C12—C13	1.368 (5)
C4—H4	0.9300	C12—N3	1.489 (5)
C5—C6	1.355 (5)	C13—C14	1.384 (5)
C5—H5	0.9300	C13—H13	0.9300
C6—C7	1.514 (5)	C14—C15	1.375 (5)
C7—S1	1.823 (5)	C14—H14	0.9300
C7—H7A	0.95 (4)	C15—H15	0.9300
C7—H7B	0.91 (4)	N1—N2	1.383 (4)
C8—N1	1.324 (4)	N1—H1A	0.93 (3)
C8—S2	1.676 (4)	N3—O1	1.208 (4)
C8—S1	1.735 (4)	N3—O2	1.212 (4)
			
C6—C1—C2	121.3 (4)	N2—C9—H9	119.6
C6—C1—H1	119.3	C10—C9—H9	119.6
C2—C1—H1	119.3	C11—C10—C15	119.7 (4)
C3—C2—C1	120.2 (5)	C11—C10—C9	118.9 (4)
C3—C2—H2	119.9	C15—C10—C9	121.3 (4)
C1—C2—H2	119.9	C10—C11—C12	118.9 (4)
C4—C3—C2	119.3 (5)	C10—C11—H11	120.6
C4—C3—H3	120.3	C12—C11—H11	120.6
C2—C3—H3	120.3	C13—C12—C11	122.5 (4)
C3—C4—C5	120.5 (5)	C13—C12—N3	118.9 (4)
C3—C4—H4	119.8	C11—C12—N3	118.5 (4)
C5—C4—H4	119.8	C12—C13—C14	117.8 (4)
C6—C5—C4	120.8 (4)	C12—C13—H13	121.1
C6—C5—H5	119.6	C14—C13—H13	121.1
C4—C5—H5	119.6	C15—C14—C13	120.8 (4)
C1—C6—C5	117.9 (4)	C15—C14—H14	119.6
C1—C6—C7	120.6 (5)	C13—C14—H14	119.6
C5—C6—C7	121.6 (5)	C14—C15—C10	120.2 (4)
C6—C7—S1	106.9 (3)	C14—C15—H15	119.9
C6—C7—H7A	113 (2)	C10—C15—H15	119.9
S1—C7—H7A	104 (2)	C8—N1—N2	119.6 (4)
C6—C7—H7B	116 (3)	C8—N1—H1A	121 (2)
S1—C7—H7B	104 (3)	N2—N1—H1A	119 (2)
H7A—C7—H7B	113 (4)	C9—N2—N1	116.5 (4)
N1—C8—S2	120.9 (3)	O1—N3—O2	124.9 (5)
N1—C8—S1	114.5 (3)	O1—N3—C12	117.6 (4)
S2—C8—S1	124.6 (3)	O2—N3—C12	117.5 (5)
N2—C9—C10	120.9 (4)	C8—S1—C7	102.0 (2)
			
C6—C1—C2—C3	0.8 (8)	N3—C12—C13—C14	179.9 (4)
C1—C2—C3—C4	−0.1 (8)	C12—C13—C14—C15	0.1 (7)
C2—C3—C4—C5	−1.0 (8)	C13—C14—C15—C10	−0.4 (6)
C3—C4—C5—C6	1.4 (8)	C11—C10—C15—C14	1.7 (6)
C2—C1—C6—C5	−0.4 (7)	C9—C10—C15—C14	−178.3 (3)
C2—C1—C6—C7	178.1 (4)	S2—C8—N1—N2	177.2 (2)
C4—C5—C6—C1	−0.7 (7)	S1—C8—N1—N2	−2.9 (4)
C4—C5—C6—C7	−179.2 (4)	C10—C9—N2—N1	177.3 (3)
C1—C6—C7—S1	96.1 (5)	C8—N1—N2—C9	178.3 (3)
C5—C6—C7—S1	−85.4 (5)	C13—C12—N3—O1	174.2 (4)
N2—C9—C10—C11	179.7 (3)	C11—C12—N3—O1	−4.7 (6)
N2—C9—C10—C15	−0.3 (5)	C13—C12—N3—O2	−6.3 (6)
C15—C10—C11—C12	−2.8 (5)	C11—C12—N3—O2	174.8 (4)
C9—C10—C11—C12	177.2 (3)	N1—C8—S1—C7	−177.5 (3)
C10—C11—C12—C13	2.6 (6)	S2—C8—S1—C7	2.4 (3)
C10—C11—C12—N3	−178.5 (3)	C6—C7—S1—C8	173.1 (4)
C11—C12—C13—C14	−1.3 (6)		

### Hydrogen-bond geometry (Å, °)

**Table:**

*D*—H···*A*	*D*—H	H···*A*	*D*···*A*	*D*—H···*A*
N1—H1A···S2^i^	0.93 (3)	2.45 (4)	3.365 (4)	170 (3)

Symmetry codes: (i) −*x*+2, −*y*+1, −*z*+1.
